# Effects of social information on life history and mating tactics of males in the orb‐web spider *Argiope bruennichi*

**DOI:** 10.1002/ece3.3672

**Published:** 2017-11-28

**Authors:** Anna‐Lena Cory, Jutta M. Schneider

**Affiliations:** ^1^ Zoologisches Institut Universität Hamburg Hamburg Hamburg Germany

**Keywords:** developmental plasticity, female availability, male mate choice, monogyny

## Abstract

Informed mating decisions are often based on social cues providing information about prospective mating opportunities. Social information early in life can trigger developmental modifications and influence later mating decisions. A high adaptive value of such adjustments is particularly obvious in systems where potential mating rates are extremely limited and have to be carried out in a short time window. Males of the sexually cannibalistic spider *Argiope bruennichi* can achieve maximally two copulations which they can use for one (monogyny) or two females (bigyny). The choice between these male mating tactics should rely on female availability that males might assess through volatile sex pheromones emitted by virgin females. We predict that in response to those female cues, males of *A. bruennichi* should mature earlier and at a smaller body size and favor a bigynous mating tactic in comparison with controls. We sampled spiders from two areas close to the Southern and Northern species range to account for differences in mate quality and seasonality. In a fully factorial design, half of the subadult males from both areas obtained silk cues of females, while the other half remained without female exposure. Adult males were subjected to no‐choice mating tests and could either monopolize the female or leave her (bigyny). We found that Southern males matured later and at a larger size than Northern males. Regardless of their origin, males also shortened the subadult stage in response to female cues, which, however, had no effects on male body mass. Contrary to our prediction, the frequencies of mating tactics were unaffected by the treatment. We conclude that while social cues during late development elicit adaptive life history adjustments, they are less important for the adjustment of mating decisions. We suggest that male tactics mostly rely on local information at the time of mate search.

## 1 INTRODUCTION

Plasticity in mating strategies is beneficial under unpredictable conditions, but it requires collecting and processing reliable information (Dall, Giraldeau, Olsson, McNamara, & Stephens, 2005; Ghalambor, McKay, Carroll, & Reznick, 2007; Gross, 1996; Schmidt, Dall, & van Gils, 2010; Sinervo & Lively, 1996; Taborsky & Brockmann, 2010). While some mating decisions immediately follow on the receipt of information, other plastic responses rely on experience during development and have a delayed effect on mate preferences or sexually selected traits (Guevara‐Fiore, 2012; Hebets, 2003; Jennions & Petrie, 1997; Snell‐Rood, 2013; Verzijden et al., 2012; West, King, & White, 2003; West‐Eberhard, 2003). Recent evidence shows that adult phenotypes including mating strategies are affected by the early social environment and suggests that social cues from conspecifics can provide young individuals with information about population densities or prospective dynamics of mates and rivals (Bailey, Gray, & Zuk, 2010; Clark et al., 2015; Guevara‐Fiore, 2012; Kasumovic & Brooks, 2011; Kodric‐Brown, 1986; Stoffer & Uetz, 2015).

Estimating the degree of future competition is particularly relevant for males in early life stages if morphological or physiological traits are plastically adjusted. Some studies showed that males reared under high densities needed more time to mature but grew larger or even developed larger testes (Gage, 1995; Kasumovic, Bruce, Herberstein, & Andrade, 2009; Kasumovic, Hall, Try, & Brooks, 2011; Stockley & Seal, 2001). Both traits can improve reproductive success through advantages in either sperm competition or physical male competition (Nylin & Gotthard, 1998). However, the trade‐off between time and size at maturation should be balanced in favor of developmental speed in scramble competition mating systems, where more time to grow will be traded against the risk of missing mating opportunities. Here, pheromonal cues produced by females that inform males about the availability of mature females can be highly fitness relevant for the timing of maturation and mating strategies (Kasumovic & Andrade, 2006; Neumann & Schneider, 2016). For instance, studies on pipefish and guppies showed that male choosiness vanishes under low female availability (Barrett, Evans, & Gasparini, 2014; Berglund, 1994). Accordingly, we expect that female cues may not only affect the ontogeny but also later mating decisions such as mating rates and male mate choice.

**Figure 1:**
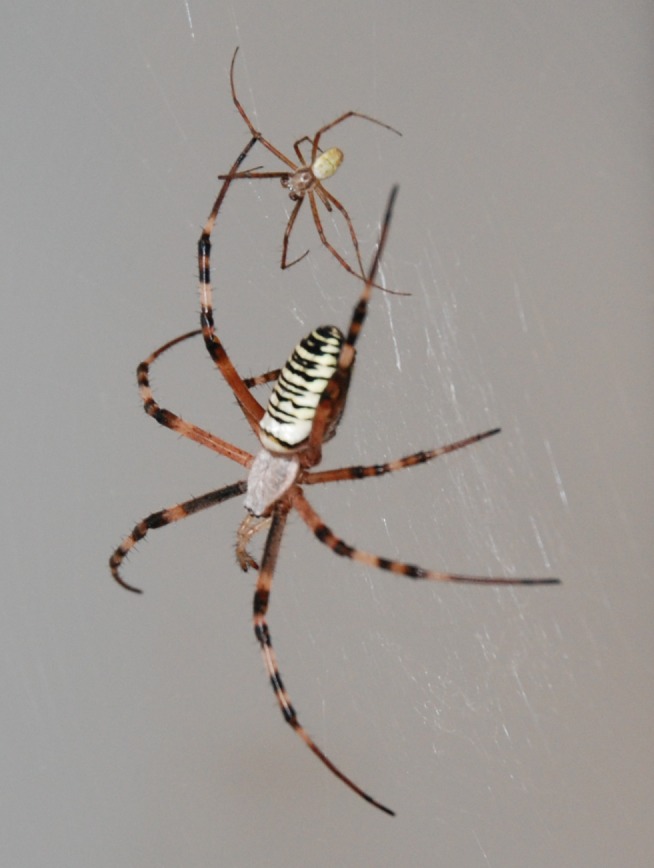
Picture of a female (large) and male (small) *Argiope bruennichi,* illustrating the extreme sexual size dimorphism. Photo by Anna‐Lena Cory

Here, we are concerned with male responses to social cues related to female availability in mating systems with extreme male mating investment. Mating systems in which males invest in monopolizing paternity with one female at the cost of future mating have been shown to evolve under a male‐biased sex ratio (Schneider & Fromhage, 2010). Such monogynous mating systems are rare but evolved several times independently in spiders (Schneider & Fromhage, 2010). Monogynous spider males plug female genital openings with parts of their genitalia (pedipalps) which cannot be reused and thereby limit their mating frequencies drastically (Fromhage & Schneider, 2006; Knoflach & van Harten, 2001; Masumoto, 1993; Nessler, Uhl, & Schneider, 2007; Schneider, Thomas, & Elgar, 2001; Schwartz, Wagner, & Hebets, 2013). As males have two pedipalps, they can achieve a maximum of two copulations. Females have two independent genital openings each connected to a different spermathecae but a male can only inseminate and plug one during any copulation. Accordingly, a virgin female that receives only one copulation retains her virginity on the unmated side. While some species practice obligate monogyny and always invest maximally in a single female, males of other species may also mate with two females (bigynous mating tactic) (Fromhage, McNamara, & Houston, 2008). Fromhage and Schneider (2012) investigated the conditions under which each mating tactic is preferred using a state‐dependent dynamic game model. Model assumptions derived from the well‐studied spider *Argiope bruennichi*, a sexually dimorphic species (Figure 1) with high rates of sexual cannibalism and very effective mate plugging by genital mutilation (Fromhage, Uhl, & Schneider, 2003; Nessler et al., 2007). The model revealed that both mating tactics can stably coexist and that bigyny should increase in frequency under a high density of virgin females that vary in quality, which may be the case early in the mating season (Fromhage & Schneider, 2012). Thus, monogyny versus bigyny is also a form of mate choice whereby bigynous males do not maximally invest in females of low quality but search for a second mating partner. The bigynous tactic involves the risk of losing paternity to rivals that inseminate the still virgin sides of both females. Despite high densities, bigynous males risk failure of finding a second virgin mating partner because mated females that are already plugged offer no paternity gains anymore. Hence, adult males will benefit from information about the availability of virgin females rather than general female density, and the perception of mating partners should affect their mating tactic.

In this study, we specifically address whether the perception of virgin female availability before maturation affects male life history and mating decisions. Spiders, including *A. bruennichi*, are an ideal system to test responses to cues because females produce volatile sex pheromones that are emitted from the female body and their silk (Chinta et al., 2010; Gaskett, 2007; Schulz, 2013). These chemical cues can be easily applied, and males perceive them from a distance. In many spiders, females stop signaling and lose receptivity after they have mated (Thomas, 2011). Also in *A. bruennichi,* the sex pheromone is only found in virgin females (Chinta et al., 2010) and in line with this, males prefer virgin over mated females (Schulte, Uhl, & Schneider, 2010). Thus, males benefit from anticipating the period when the density of virgin females is highest. The mating season lasts for nearly a month, although most mating events occur within 2 weeks when the majority of females molt to maturity (Zimmer, Welke, & Schneider, 2012). Males start maturing earlier than females and soon leave their webs to actively search for females. They can maximally copulate twice and generally die during their second copulation, although many do not survive their first copulation due to female attacks (Schneider, Fromhage, & Uhl, 2005). These spiders can occur in high densities and virgin females access quickly to mating. Accordingly, population dynamics can quickly change during the mating season and males should be very sensitive to female cues to not miss the optimal timing for mate search.

*Argiope bruennichi* experienced a recent range expansion from Mediterranean into Northern Europe. To account for potential differences in ecology and life history that may have led to local adaptations and also may have affected decision rules underlying mating tactics, we compared individuals from Northern and Southern Europe representing the edges of the species distribution (Krehenwinkel, Rodder, & Tautz, 2015; Krehenwinkel & Tautz, 2013). Northern males experience a shorter season, which presumably synchronizes maturation and makes female availability more predictable, but restricts the time for growth in both sexes. Indeed, Northern individuals are smaller than individuals from the south (Krehenwinkel & Tautz, 2013), and the females produce fewer eggs because fecundity is a direct function of body size (Marshall & Gittleman, 1994; Simpson, 1995). The reduced female quality, as well as the higher seasonality in the north, should have selected for higher rates of bigyny in Northern males. During a field study in Northern Germany, the frequency of bigyny was estimated to be 50% (Welke, Zimmer, & Schneider, 2012). Should bigyny be affected by female size and by unpredictable female availability, we expect lower rates of bigyny in Southern males.

To test our predictions, we sampled egg sacs or recently hatched spiderlings from different locations in Europe and raised them under standardized conditions. Half of the male spiders received silk from virgin females as cues, while the other half got no information. To receive quantifiable life history traits, we assessed the time that males needed for their last instar and measured the body size of males shortly after maturation. After reaching sexual maturity, males were allowed either to mate twice with the same female (monogyny) or to leave the female after the first mating (bigyny).

To sum up our hypotheses, we predict that without information through pheromonal cues produced by females, (1a) Northern males need less time to develop because they are smaller and that (1b) they are more often bigynous than Southern males because females are of lower quality and the high seasonality should reduce uncertainty about female availability. In the presence of female cues during development, we hypothesize that (2a) both, Northern and Southern males, have a shorter developmental time compared with uninformed males. Furthermore, we hypothesize that (2b) informed males engage more often in a bigynous tactic. If the frequency of bigyny is influenced by female cues, it might also inform males about the opportunity to choose. Thus, we predict that (3) informed males are choosier, which should become apparent through an interaction between the exposure of female cues and female quality such that there is an effect of female size on the mating tactic.

## 2 MATERIAL AND METHODS

### 2.1 Collection and maintenance

We collected spiders from two different geographical areas in Northern and Southern Europe between 25 April and 18 May 2015. In the Northern area, we collected 26 egg sacs shortly before the spiderlings left their egg sac at four different locations (Northern Germany: Dahlenburg, Wedel, Lüneburg, and Buxtehude). In the Southern area, we collected young juvenile spiders having had one or two molts outside their egg sacs. The Southern spiders also came from four different locations (Belfou, Carcassonne, Villefloure, and Gardie/Saint Hillaire). *Argiope bruennichi* is abundant in Europe and the collection of these spiders required no permits.

In the laboratory, spiders were raised under ambient temperatures in upturned plastic cups of different volumes depending on the spiders’ size. We provided them with water six times a week. Twice per week, juvenile spiders and males received approximately 20 *Drosophila* spec. and large females received three *Calliphora* spec. We checked for molts to subadult and adult stage at least six times a week. The subadult stage is the last developmental stage before the final molt. Subadult and adult males can be recognized by their secondary genital organs, the pedipalps. In subadult males, the distal part of the pedipalps is thickened and only shows differentiated sclerite structures if males are sexually mature. Adult females can be recognized by inspecting their genitalia for the presence of the scape which covers the genital openings (Uhl, Nessler, & Schneider, 2007). Adult females were transferred into Perspex frames (approx. 35 × 35 × 6 cm) for building orb webs in which the mating tests were conducted. About half of the males died during the mating tests because of sexual cannibalism or spontaneous male death, the others mated only once and died of old age. Females either died naturally in the laboratory or were frozen at −80°C after they had laid two egg sacs which is the average number in the laboratory (Schneider et al., 2005).

### 2.2 Provision of female cues

**Figure 2:**
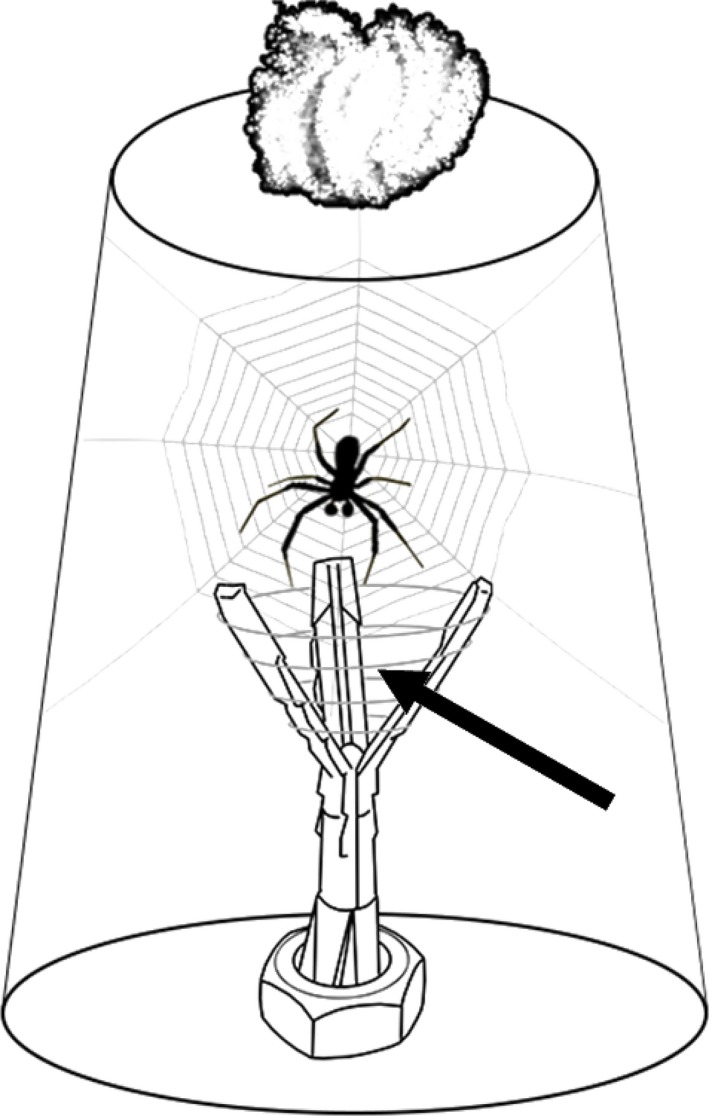
Scheme of a juvenile male in a typical upturned plastic cup and a silk carrier. Silk from a virgin female's web is wrapped around the top of the silk carrier (dark gray lines, arrow). The cups had a hole in the bottom fitted with cotton wool which was regularly moistened with water

Shortly after reaching the subadult stage, males were split into two treatments of which one was sheltered from female pheromones inside a walk‐in climate chamber (Southern males: *N* = 44, Northern males: *N* = 57). The other half was kept in an identical climate chamber and received female cues (Southern males: *N* = 45, Northern males: *N* = 59). Both climate chambers had a photoperiod of 14:10 LD, a temperature of 23°C and 50% humidity at daytime and 20°C and 70% humidity at night. Female cues were given in the form of silk taken from the web of a virgin female. Silk was wrapped around a self‐made construction (*silk carrier*) consisting of a raw plug and an adjustable nut (Figure 2). With the head of the *silk carrier*, we wrapped silk of the sticky spiral that was located between two adjacent radii. Each male cup was equipped with a *silk carrier* including the males without female silk to control for undesired effects caused by the *silk carriers* themselves. We ensured that males had regular contact with the silk, at least with their forelegs and pedipalps, which are known to be important for the reception of sex pheromones (Jiao, Chen, Du, Chen, & Liu, 2011). Depending on environmental conditions, pheromones lose their activity which can be after a few weeks but also after a few days (Baruffaldi, Costa, Rodriguez, & Gonzalez, 2010). To ensure that males had constant access to female pheromones, we replaced the *silk carriers* of both treatment groups three times per week with 1–2 days between replacements. All males received female cues from both Northern and Southern females. Females were virgins and not older than 16 days postmaturation, and males received cues of the same female maximally twice. After reaching maturity, males received female cues or empty *silk carriers* for six more days. After that, males from the treatment with cues of females were transferred to the climate chamber with the males without female cues and stayed there until they were used for mating tests.

We used silk of virgin females that were born and raised in our laboratory. The parental lineage of these females was collected in 2014, mostly at the same locations as in 2015. Females that provided silk were not used for mating tests. As females are larger than males, they need more time to develop. To constantly have adult females for silk provisioning at hand, we opened some egg sacs already in March (instead of waiting for natural hatching in May) and transferred large juveniles to a cabinet with a temperature of 27°C at daytime and 20°C at night and a photoperiod of 14:10 LD with long days to speed up their development. Adult females were kept in the same climate chamber, in which the males receiving female cues were raised.

#### 2.2.1 Mating tests

The mating tests were conducted in spatially divided shelf compartments (55 × 75 × 67 cm) that were only open at the front to keep potential pheromone transmission between mating tests as low as possible. The front had to be open to be able to interfere during copulation (see below). The experimental set‐up consisted of three Perspex frames. The test frame with a female in her web was positioned in the front, and two frames with empty webs were positioned next to the test frame at an angle of 90°, forming a U‐shape. Thus, males could escape from the test frame into an empty and safe frame in two directions.

Males of both geographical origins were randomly chosen from both treatments to mate with females of the same geographical origin. A mating trial began by placing a male gently into the left or right upper corner of the female web with a paintbrush. From this corner position, males would enter the web and signal their presence and reveal themselves as a mating partner (Wignall & Herberstein, 2013). Our main interest was to determine whether males would use a monogynous or bigynous mating tactic. However, male mating decisions are often veiled due to female aggression and sexual cannibalism. If males died after the first copulation, we could not be certain whether the males sacrificed themselves or whether they were not fast enough to escape the female attack. We, therefore, prevented sexual cannibalism after the first copulation following a standardized scheme. In *A. bruennichi*, the copulation is short but easy to recognize because the female bends over and pulls silk out of her spinnerets within the second of genital contact. During the tests, we intervened 2‐s after this and quickly placed a toothpick between the female chelicerae, giving the males a chance to escape. Although we blocked the female chelicerae, females still tried to wrap males into silk. In the process of freeing themselves from the silk wrapping males regularly lost legs as common in undisturbed copulations. Males that lose 1–3 legs can still accomplish further mate search and a second copulation, but we excluded four tests in which males had lost all four forelegs. We further excluded three Southern males and five Northern males that did not try to escape from the female and died during the first copulation. All other males survived and did not copulate longer than known from other studies: 91.5% of males copulated less than 10 s (*N* = 106), which is the threshold after which males are generally cannibalised (Fromhage et al., 2003; Nessler, Uhl, & Schneider, 2009). Note that we measured the copulation duration while we disturbed the female attack. As these measures might not be accurate, we did not use them in the final statistics, although exploratory statistics showed no influence. After the first copulation, we monitored whether the males copulated again (monogyny) or whether they left the female (bigyny). We defined “leaving a female” when a male left the test frame, moved a distance of at least 18 cm (half frame length), and stayed away for at least one hour. We also checked that the male had no contact‐thread to the female web. Some males left the shelf compartment, forcing us to interrupt the trial to recover the male. Experiments were conducted between 9 am and 5 pm after which the shelf compartment was sealed until the next morning. The mating strategy of single‐mated males that had not re‐mated during the first day could be assessed by inspecting the set‐up on the next morning: A male was considered monogynous if his body remains were found underneath the female web which is good evidence for sexual cannibalism after copulation. A male was considered bigynous if he had not made contact to the test‐female's web (no draglines visible) but was sitting in one of the other frames. We excluded one male because he died the next day, which might be a sign of injury.

#### 2.2.2 Measurements

Adult males and females were weighed to an accuracy of 0.1 mg on the day of sexual maturity and again on the test day, which was between 13 and 25 days (median = 18 days) after reaching maturity. We measured the tibia‐patella length of both forelegs from frozen females and used leg size as an approximation for the overall spider size. To estimate female condition, we used leg size as an approximation for the overall body size (e.g., Higgins, 1992) and computed the residuals of the regression between leg size and test weight. We refrained from measuring leg length in males because some monogynous males were cannibalised over night by the female so that no body parts were left to measure which would have led to a reduced sample size. Male body size and body weight at maturation are highly correlated (Schulte et al., 2010) and we, therefore, used weight as a proxy for size at maturity. Without the leg length of males, we could not estimate the male condition in the same way as we estimated the female condition. Instead, we used an approximation for changes in male energy reserves and calculated the relative weight difference between adult weight and test weight, which was generally negative because males rarely feed after reaching sexual maturity.

### 2.3 Statistical analysis

We analyzed our data within the R environment (R Developmental Core Team, 2014). To test for differences in the homogeneity of variance between groups, we performed *F* tests and Levene tests (part of the package “car”) (Fox & Weisberg, 2011). As we had to ignore potential dependence structures, these results should be interpreted with some caution. Moreover, we performed statistical modeling to test for population differences and for effects of female cues on developmental time and male mating tactics. Effects of female cues were analyzed for both geographical origins separately. We used the R‐package “fitdistrplus” to evaluate error structures and log‐transformed the data to have a normal error structure if necessary (Delignette‐Muller & Dutang, 2015). To test our models, we used the R‐package “nlme” (Pinheiro, Bates, Debroy, Sarkar, & Team, 2013). We simplified the models by removing fixed variables using the anova command (maximum likelihood methods) and compared the prior model with the simplified model.

#### 2.3.1 Developmental differences between individuals of different geographical origin

We compared the duration of the subadult stage (in days) and the male adult weight of Northern (*N* = 116) and Southern males (*N* = 89) by performing linear mixed effects models with “geographical origin” as fixed factor. We included “*collection site*” as a random factor because we found differences in the duration of the subadult stage between the four collection sites in each geographical origin (Kruskal‐Wallis test: Southern males: *N* = 89, Chi² = 12.44, df = 3, *p* = .006; Northern males: *N* = 116, Chi² = 10.47, df = 3, *p* = .015).

If Southern males experience a more variable mating season as we assumed, the timing of male maturation might also be more variable and less synchronous in Southern compared to Northern populations. To test this, we conducted a linear mixed effect model and used as response variable the date of the first adult Northern or Southern male as the starting point of the mating season and counted the days that passed between the starting points and the adult dates of all males. As explanatory variables, we included the “*geographical origin*” as fixed factor and the “*collection site*” as random factor.

#### 2.3.2 Effects of female cues on developmental time

We constructed models in which we tested whether the reception of female cues had a significant effect on male developmental time (here duration of the subadult stage). In addition to the reception of females cues (yes, no), we used the adult weight of males as a continuous explanatory variable because a prolonged subadult stage may lead to a larger body size. For the analysis of Southern males, we included “*collection site*” as a random factor. Remember that we collected egg sacs in Northern populations and, thus, had to use siblings. Therefore, we included the “*family lineage*” as a random factor in the model with Northern spider data. As we analyzed both populations separately, we also checked whether in Northern males the “*family lineage*” had an effect on the duration of the subadult stage and indeed found differences in the developmental time between families (only families with at least three replicates were included Kruskal‐Wallis test: *N* = 105, df = 17, Chi² = 41.904, *p* = .001).

#### 2.3.3 Effects of geographical origin and female cues on male mating tactics

To test whether the addition of female cues during the subadult stage would influence later mating decisions, we conducted binary logistic models with the two mating tactics (bigyny, monogyny) as the response variable. We did not use “*collection site*” or “*family lineage*” as a random factor because, in both populations, the estimate of the variance explained by these random factors was 0. As explanatory variables, we included reception of female cues, female size, and body condition, the relative weight change of males, female and male postmature age, and the duration of the subadult stage. Moreover, we included into the model the interaction term “*female cues: female size*” to test whether the reception of female cues would influence male choosiness. Additionally, we considered the interaction between *female cues* and *relative weight change in males* because we found a significant influence of male condition as a single variable on mating decisions.

We had to exclude three tests because we could not measure the legs of two females and found a measuring error in one male. In the end, we had a sample size of 50 Northern males with 25 males in each treatment group and 56 Southern males of which 26 males received female silk.

To test whether males of both treatment groups had similar conditions during the mating tests, we tested for differences in the explanatory variables between the treatment groups within the model. After we had to reduce the sample size, we found in Southern males that the males receiving female cues had a shorter subadult stage (Mann‐Whitney *U* Test: *N* = 56, *W* = 511, *p* = .046) and were younger (Mann‐Whitney *U* test: *N* = 56, *W* = 561.5, *p* = .004). We tested whether this would have an impact on our analysis, but found no evidence.

## 3 RESULTS

### 3.1 Developmental differences between individuals of different geographical origins

**Figure 3:**
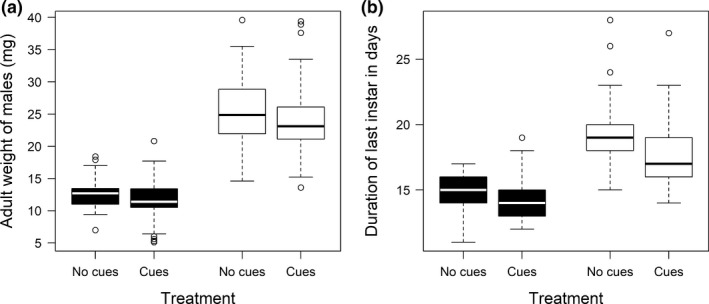
Treatment effects (no cues or cues) on adult weight (a) and the duration of the subadult instar (b) in Northern (black) and Southern (white) males

Southern males matured at a larger size than Northern males (Linear mixed effect model: *N* = 204, *W* = 99.5, *p* < .001; Figure 3a) and needed more time to reach sexual maturity (Linear mixed effect model: *N* = 204, *L* Ratio = 16.37, *p* < .0001; Figure 3b). The variation in adult weight (Levene test: *N* = 204, *df* = 1, *F* = 31.472, *p* < .001) and in developmental time (Levene test: *N* = 204, *df* = 1, *F* = 16.806, *p* < .001) was heterogeneous between geographical origins with the range and interquartile range of both traits being larger and more uneven in Southern males than in Northern males (Figure 3a, b).

We presumed that Southern males are adapted to a longer mating season, which should be revealed by a larger variation in the timing of maturation than in Northern males. However, Southern (15.4 ± 5.8 days; range: 32 days) and Northern males (13.3 ± 6.6 days; range = 30 days) matured within a similar timeframe (Linear mixed effect model: *N* = 204, *df* = 1, *r*‐ratio = 1.871, *p* = .171) and variances did not differ significantly (*F* test: *N* = 204, *F*(114, 88) = 1.305, *p* = .1924) even though the first Southern male had his final molt 6 days before the first Northern male.

### 3.2 Effects of female cues on developmental time

Analyzing both geographical origins separately, we found that the reception of female cues consistently led to a significant reduction in the developmental time (Table 1) with homogeneous variation between males of both treatment groups (*female cues*/*no female cues*) (Northern males: Levene test: *N* = 116, *df* = 1, *F* = 0.571, *p* = .452; Southern males: Levene test: *N* = 89, *df* = 1, *F* = 0.301, *p* = .585; Figure 3b). However, we found no support for the prediction that a reduction in developmental time led to a reduced adult weight in males. In line with this finding, there was no relationship between the adult weight and developmental time (Table 1) nor did the reception of cues lead to different variance in adult weight between treatment groups (Northern males: Levene test: *N* = 116, *df* = 1, *F* = 2.175, *p* = .143; Southern males: Levene test: *N* = 89, *df* = 1, *F* = 0.497, *p* = .483; Figure 3a).

**Table 1:** Results from linear mixed effect models on the developmental time (duration of the subadult instar) of Northern and Southern males

Variable		Estimate ± *SE*	*L* ratio	*df*	*p*
Northern ♂♂ (*N* = 116)					
Intercept	Reference level: no ♀ cues	(15.097 ± 1.068)14.978 ± 0.233			
Cues (♀ cues; no ♀ cues)	♀ cues	(−1.348 ± 1.246)−0.566 ± 0.254	4.929	1	.026*
♂ adult weight		(−0.009 ± 0.082)	0.488	1	.485
Cues ♂ adult weight		(0.065 ± 0.099)	0.428	1	.513
Southern ♂♂ (N = 89)					
Intercept	Reference level: no ♀ cues	(3.011 ± 0.096)2.943 ± 0.032			
Cues (♀ cues; no ♀ cues)	♀ cues	(0.020 ± 0.128)−0.058 ± 0.028	4.284	1	.039*
♂ adult weight		(−0.003 ± 0.004)	2.853	1	.091
Cues : ♂ adult weight		(−0.003 ± 0.005)	0.442	1	.506

Data from Southern males (estimates and *SE*) were log‐transformed. In Northern males, we used family lineage as a random factor (26 groups) and in Southern males the collection site (4 groups). Estimates and *SE*s of the full models are in brackets; those of the minimal adequate model are without brackets. The *L* ratio is the abbreviation for the likelihood ratio. Significant effects are indicated by asterisks.

### 3.3 Effects of geographical origin and female cues on male mating tactics

**Figure 4:**
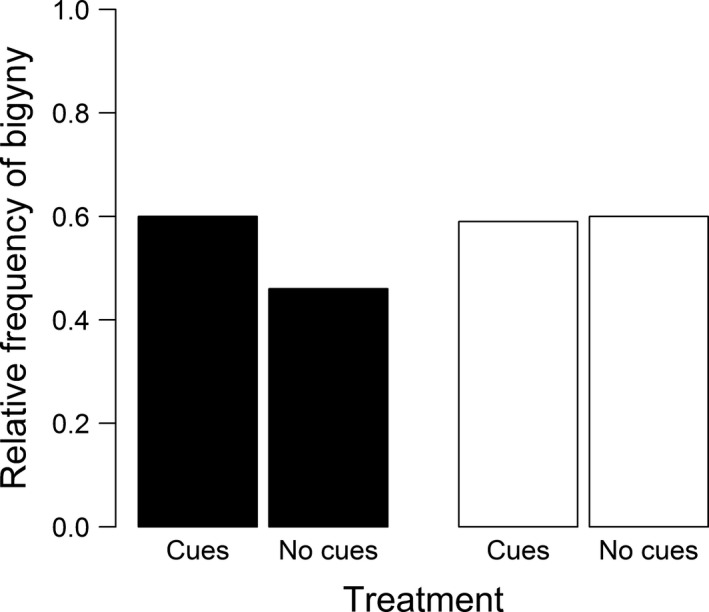
Relative frequency of bigyny depending on the reception of female cues by Northern (black bars) and Southern (white bars) males

**Figure 5:**
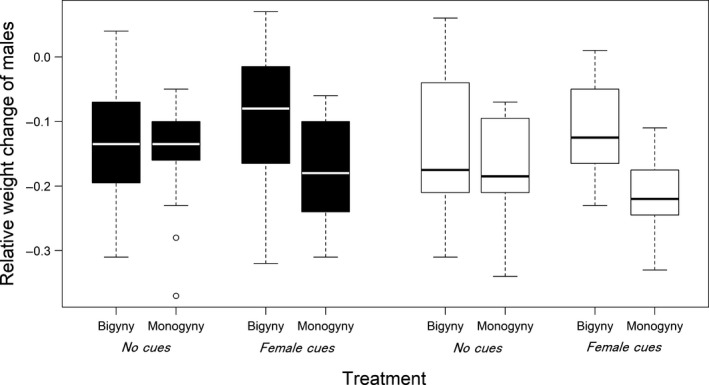
Descriptive statistics of the relative weight changes between adult molt and mating separated for males that selected the bigynous and monogynous mating tactic and received female cues or not. Black bars depict data from Northern males and white bars from Southern males

**Figure 6:**
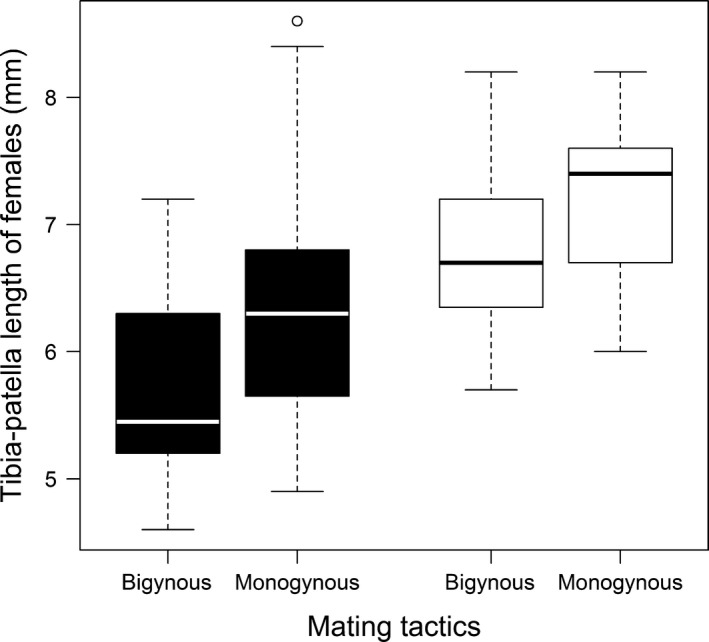
Males were more likely monogynous if the female had a larger body size. Tibia‐patella length was used as an approximation for overall female size. Black bars depict data from Northern males and white bars show Southern males

The frequency of the bigynous mating tactic was 56% and did not differ between Northern and Southern males (binary logistic regression: *N* = 109, DF‐deviance = 0.348, *p* = .2369; Figure 4). The treatment (*female cues*/*no female cues*) as factor or in the interaction with female size did not affect the frequency of bigyny (Table 2, Figure 4). Instead, we found a significant interaction between the treatment and the relative weight change in Southern males (Table 2). Southern males with a relatively low weight change were more likely bigynous but only if they had received female cues (Figure 5). Northern males that lost relatively less weight also favored the bigynous tactic (Figure 5), but this was independent of the treatment (Table 2). The second significant effect on the probability of bigyny was female size. Corroborating findings from a field study (Welke et al., 2012) males were more likely bigynous when the female was small (Table 2; Figure 6), although this was not significant (*p* = .056) in Southern males. Neither the female postmaturation age nor female condition had a significant effect (Table 2).

**Table 2:** Summary of results about effects on the probability of monogynous and bigynous mating tactics

Effect		Estimates ± *SE*	*df*‐deviance	*df*	*p*
Northern ♂♂ (N = 50)					
Intercept	(Reference level: no ♀ cues)	(−17.008 ± 8.285) −6.589 ± 2.620			
Cues (♀ cues; no ♀ cues)	♀ cues	(0.180 ± 6.197)	0.323	1	.570
♀ size		(0.867 ± 0.724) 0.924 ± 0.411	6.101	1	.014*
♀ condition		(1.471 ± 1.834)	1.379	1	.240
♀ postmature age		(0.091 ± 0.273)	0.151	1	.698
Rel. ♂ weight change		(−6.674 ± 6.258) −8.193 ± 3.711	5.944	1	.015*
♂ postmature age		(0.196 ± 0.131)	1.974	1	.160
Duration of subadult stage		(0.485 ± 0.317)	2.109	1	.147
Cues : ♀ size		(−0.149 ± 0.979)	0.038	1	.845
Cues : rel. ♂ weight change		(−1.963 ± 8.146)	0.023	1	.879
Southern ♂♂ (*N* = 56)					
Intercept	(Reference level: no ♀ cues)	(0.834 ± 5.495) −0.920 ± 0.756			
Cues (♀ cues; no ♀ cues)	♀ cues	(−17.622 ± 12.433) −3.369 ± 1.903	–	–	–
♀ size		(0.949 ± 0.743)	3.651	1	.056
♀ condition		(−0.470 ± 2.027)	0.039	1	.843
♀ postmature age		(0.120 ± 0.237)	0.606	1	.436
Rel. ♂ weight change		(−3.264 ± 4.663) −3.231 ± 4.033	–	–	–
♂ postmature age		(−0.066 ± 0.119)	0.275	1	.600
Duration of subadult stage		(−0.314 ± 0.190)	3.466	1	.063
Cues : ♀ size		(1.482 ± 1.419)	1.308	1	.253
Cues : rel. ♂ weight change		(−37.578 ± 20.851) −19.875 ± 10.392	7.748	1	.005*

The estimates and standard errors of the estimates are logit‐transformed because they arose from a binary logistic regression. The estimates and the standard errors of the full model are in brackets, and the results of the minimal adequate model are without brackets. Significant effects are indicated by asterisks.

## 4 DISCUSSION

Plastic traits, as for example life history strategies or alternative mating tactics, are known to respond to cues that provide social information (Engqvist & Taborsky, 2017; Kasumovic & Brooks, 2011; Kodric‐Brown, 1986; Rodriguez, Rebar, & Fowler‐Finn, 2013). Upon maturation, females of *Argiope bruennichi* emit a volatile sex pheromone that attracts males (Chinta et al., 2010). We tested whether the reception of female pheromones during the subadult stage influences life history and mating decisions in males. In a common garden situation, we compared males that came from the Northern and Southern edges of the species’ distribution. These regions differ in climatic factors that likely affect the available duration for growth and reproduction, and indeed, males from Northern populations had a shorter developmental time and were smaller than males from Southern populations. Males from both regions matured earlier if they received female cues during the subadult stage. While this provides evidence that males indeed perceived the silk cues, we found no corresponding differences in the frequencies of monogynous and bigynous mating tactics. Whether or not a Southern male selected the bigynous tactic depended on his body condition in interaction with the treatment whereas for Northern males female quality was more important than the presence of cues.

Ecological differences may lead to local adaptations in traits under natural and sexual selection (Jennions & Petrie, 1997; Stearns, 2000). During their northwards range expansion (Krehenwinkel & Tautz, 2013; Krehenwinkel et al., 2015), *A. bruennichi* had to adapt to a shorter spring and summer and a shortening of the mating season. As expected, we found that Southern males had a longer developmental time and matured at a larger body size compared to Northern males even though they developed under the same conditions in the laboratory. We had also expected a larger variation in these traits in Southern compared to Northern males, but males from both regions showed a similar maturation window of about 30 days. This pattern might suggest that the trade‐off between age and size at maturation is generally shifted toward time matching the notion that it is of paramount importance for male reproductive success to hit the peak of female maturation (Zimmer et al., 2012). Across all populations, the length of the subadult stage is found to be a plastic trait that responded to the presence of silk from webs of virgin females.

In agreement with two earlier studies on two other spider species (Kasumovic & Andrade, 2006; Neumann & Schneider, 2016), male *A. bruennichi* shortened their subadult stage if they were exposed to the silk of virgin females. The silk contains a specific sex pheromone that attracts males to their web and induces male courtship (Chinta et al., 2010). Our results indicate that males make use of this sex pheromone to assess the availability of virgin females and plastically adjust their developmental time to this social information. This is adaptive because mating chances are limited and male reproductive success relies on finding virgin females as they cannot gain paternity with double‐mated and plugged females (Nessler et al., 2007). A strong preference for virgin versus mated females was confirmed in simultaneous mate choice experiments (Schneider, Zimmer, Gatz, & Sauerland, 2016; Schulte et al., 2010). Differentiation between virgin and mated females likely depends on the sex pheromone which virgin but not mated females produce (Chinta et al., 2010). Our results are consistent with the mate opportunity hypothesis (Brooks et al., 2005) that males benefit from maturing in time and scramble for opportunities to be first in finding a female that has just matured in the vicinity. Indeed, the local availability of virgin females can be very heterogeneous as females mature over a period of a few weeks (Zimmer et al., 2012). Even if early virgins are taken by rivals, males benefit from catching the onset of female maturation as they may encounter and guard subadult females that mature within days (Zimmer & Schneider, 2016). These males mate with the female during the molting process when her exoskeleton is still soft and thereby avoid sexual cannibalism (Uhl, Zimmer, Renner, & Schneider, 2015). Even though it is unknown how males locate subadult females that are not known to produce sex pheromones, a proportion of 56.45% of all females has been found guarded by a male (Zimmer & Schneider, 2016).

According to life history theory, early maturation should come at a cost, which is generally presumed to be body size (Abrams, Leimar, Nylin, & Wiklund, 1996; Nylin & Gotthard, 1998) following the classical trade‐off that males either mature early and are small or mature later and are large. Compromising large body size is expected to have high fitness costs if male mating success depends on physical strength (Hunt, Breuker, Sadowski, & Moore, 2009; Jennions, Moller, & Petrie, 2001). However, in species that scramble for access to females, negative fitness consequences of reduced body size due to earlier maturation should be less severe. Contrary to life history theory the body size of *A. bruennichi* males was unaffected by earlier maturation. Previous studies on effects of pheromonal cues produced by females on maturation are inconsistent in that no effect on body size was detected in the orb‐web spider *Nephila senegalensis* (Neumann & Schneider, 2016), whereas male red‐back spiders *Latrodectus hasselti*, had a smaller body size when they matured earlier in response to female cues (Kasumovic & Andrade, 2006). A difference between the studies was the feeding regime that was ad libitum in *A. bruennichi* and *N. senegalensis* but variable in red‐back spiders (Kasumovic & Andrade, 2006; Neumann & Schneider, 2016). The size effect in red‐back spiders was primarily apparent in males that received a low diet (Kasumovic & Andrade, 2006). Future experiments should evaluate if the costs of early maturation vary depending on the food regime in the laboratory and compare the finding to responses of males collected in the field.

The plasticity in the duration of the subadult instar appears to be a highly adaptive trait in species with extremely low mating rates that crucially depend on mating with virgin females. Finding a similar response in three spider species from different families (*L. hasselti, N. senegalensis,* and *A. bruennichi*) suggests that the length of the subadult instar might be another trait that evolved independently in monogynous mating systems, next to genital damage and permanent sperm depletion as adults (Michalik & Rittschof, 2011; Uhl, Nessler, & Schneider, 2010). Its presence supports the general hypothesis that protandry is strongly favored by selection in mating systems with a first‐male advantage in sperm competition (Simmons, Llorens, Schinzig, Hosken, & Craig, 1994; Uhl et al., 2010; Wedell, 1992; Wiklund & Forsberg, 1991).

Alternative mating tactics can be conditionally adjusted to prevailing conditions, or they might be fixed due to genetic adaptations or experience during development (Taborsky & Brockmann, 2010; West‐Eberhard, 2003). Using information, for example about female availability minimizes uncertainty about the success of a bigynous tactic which depends on the chance of finding a second female. However, we have to reject our hypothesis that males adjust their mating tactic to social information received during pre‐adult stages. A previous study found that cues that males received as adults did modify mating behavior (Nessler et al., 2009). This might suggest that information about female availability during mate search is more reliable, and it might be adaptive to adjust mating tactics then. This is in accordance with the study of Swanger and Zuk (2015), who found that in the Pacific field crickets (*Teleogryllus oceanicus*), only the adult social environment shape female responses to sexual signals, but not experience during development. However, in the closely related species *T. commodus,* the quantity of male calls prior to maturity affected how quickly females choose males (Kasumovic, Hall, & Brooks, 2012). Thus, whether and how early social information affects the adult life of individuals differs between species and as shown here, even within species suggesting that local ecology plays an important role.

The success of the bigynous mating tactic should depend on the energy reserves a male has left to continue mate searching. Correspondingly, males of both populations were more likely bigynous if their relative weight loss was small. The reduction in body weight occurs because adult males search for females soon after they reach sexual maturity. From then on, males no longer build capture webs and barely eat (Foellmer & Fairbairn, 2005). Searching for a second mating partner involves additional risks and costs. Males not only need to find a second female, the search itself is risky because it is energy and time‐consuming and bears the risk of predation (Andrade, 2003; Kasumovic, Bruce, Herberstein, & Andrade, 2007). Moreover, if males have found a second female they need to perform courtship display again, which is energetically costly (Cady, Delaney, & Uetz, 2011; Stoltz, Elias, & Andrade, 2009). Therefore, it may only be adaptive to engage in a bigynous tactic if the male has enough energy reserves to master a second mating. It is, therefore, puzzling that uninformed Southern males did not show this flexibility as the factor of body weight change was only significant in combination with the presence of cues. In the absence of female cues, these males may have followed a behavioral program that makes sense very early in the season when most females are not yet mature (Nessler et al., 2009; Zimmer et al., 2012).

Bigyny versus monogyny can be viewed as a form of male mate choice (Fromhage & Schneider, 2012; Welke et al., 2012). Male mate choice is generally rare but might evolve in systems with high male mating investment (Barry & Kokko, 2010). Males should only invest in mate choice if female availability provides opportunities to choose and if variation in female quality justifies the costs of choosiness (Bonduriansky, 2001; Edward & Chapman, 2011). For example, male pipefish *Syngnathus typhle* were only choosy if the operational sex ratio was female‐biased (Berglund, 1994). In our study, males were more likely monogynous if the female was relatively large although the correlation was only significant in Northern males. This finding corroborates results from a field study in Northern Germany (Welke et al., 2012). In Southern males, female size had no influence on male mating decisions. Perhaps male selectivity according to female size only emerged in the course of the colonization of Northern Europe. A decrease in female body size and thus fecundity during the range expansion may make female quality a more relevant trait. However, as our experiments did not focus on female quality and results are correlational, we need further studies that investigate the mechanisms of quality‐dependent male mate choice and address questions concerning the ability of males to determine female size or condition by female chemical cues.

Less than 40% of males survive their first copulation making it impossible to score the mating tactic of the majority of males. Therefore, we prevented sexual cannibalism by blocking the female chelicerae with a paintbrush allowing males to escape the wrapping. We found no indications (see Material and Methods) that the handling affected male behavior or the frequency of the observed tactics. Therefore, we assume that the decision to sacrifice their life during the first copulation or to attempt an escape was made before our interference.

Our findings support the idea that the use of social information is a fitness relevant trait in variable environments because it minimizes uncertainty. The adaptation to local environmental conditions should take place in different stages of life history including the mating season. The perception of social cues can give information about the availability of mating partners and competitive conditions but also about the start of the mating or breeding season. Several studies could show that mismatches in sexual maturity and the start of the breeding season lead to reduced fitness or even to extinction (Jones & Cresswell, 2010; Reed, Jenouvrier, & Visser, 2013). The successful colonization of new habitats and the adaptation to rapidly changing climate conditions requires processing a combination of information from the abiotic and biotic environment. Our findings support this idea and additionally show that independent of climatic conditions gathering and processing information by social cues is important in sexual selection.

In conclusion, we show that *A. bruennichi* males plastically respond to their social environment through the reception of chemical cues produced by virgin females. These cues are used to make information‐based decisions on the timing of maturation, but they are less relevant for later mating decisions. This may be adaptive because population dynamics and thus female availability may change rapidly, which is why the availability during the male's subadult stage may not be the same than during mate search. A flexible use of mating tactics and the use of local information may be beneficial to respond to environmental conditions during the mating season. Perhaps this plasticity has facilitated swift adaptation to novel conditions during the recent range expansion of the species.

Behavioral and life history responses to social cues have been investigated in several insect and spider species and revealed intriguing similarities and differences between and within species. More studies are desired to explore variation in costs and benefits of life history adjustments and elucidate the selection pressures favoring the evolution of plastic responses in development and behavior to social cues.

## AUTHOR CONTRIBUTIONS

Jutta Schneider prepared the concept of the experiment, designed the experiment, interpreted the data and revised the manuscript. Anna‐Lena Cory designed the experiment, collected, analyzed and interpreted the data, and drafted and revised the manuscript.

## CONFLICT OF INTEREST

None declared.
